# New quantum physics, solving puzzles of Wheeler’s delayed choice and a particle’s passing N slits simultaneously and quantum oscillator in experiments

**DOI:** 10.1038/s41598-022-17667-1

**Published:** 2022-08-24

**Authors:** Changyu Huang, Yong-Chang Huang, Yi-You Nie

**Affiliations:** 1 Institute of Theoretical Physics, Jiangxi Normal University, Nanchang, 330022 China; 2 Lawrence Berkeley National Laboratory, 1 Cyclotron Road, Berkeley, CA 94720 USA; 3 Department of Physics and Astronomy, Purdue University, 525 Northwestern Avenue, W. Lafayette, IN 47907-2036 USA; 4 Institute of Theoretical Physics, Beijing University of Technology, Beijing, 100124 China

**Keywords:** Quantum mechanics, Theoretical physics

## Abstract

This paper discovers new quantum physics, and gives solutions to puzzles of Wheeler’s delayed choice and a particle’s passing many slits simultaneously by exact quantum physics expressions. We further show new quantum control, new quantum oscillation, new quantum control experiments and new quantum oscillator being able to be installed in quantum communication network etc. We discover that the ability of a photon to hit electrons out in photoelectric effect is complementarily equivalent to the ability of wave of a photon to simultaneously pass through many slits in wave-particle duality. Objective criterion for distinguishing classical and quantum particles is found, and this paper gives applicable realm of quantum theories and new quantum physics expressions of wave-particle duality. All these studies above should be classified as classical and quantum particles, then classical particle and quantum particle wave cannot and can pass many slits, respectively. This paper discovers wave-particle duality’s origin of displaying both wave property from plane wave part of the general Fourier expansion and particle property from the general Fourier expansion coefficients with the particle’s global property and spins etc. We give the superposition state representation of wave-particle duality, further find the collapse of the duality superposition state to wave or particle state. The collapsed wave or particle state is related to the measure of wave or particle property. Then, we explain why sometimes it's a wave or a particle. Our achieved results are truly tested, and we discover new measured attractive state and quantum wave collapse velocity expression.

## Introduction

In modern physics, the double slit experiments show that microscopic particles can display both classical particle and wave properties, and they show the probabilistic nature of phenomena of quantum mechanics^1^.

In 1801, the double slit experiment was first finished with light by T. Young^2^, after a long time, electrons display the same behavior in 1927^2^.

A coherent light source, e.g., a laser beam, lights up one plate pierced by two parallel slits, behind the plate, light of passing through the slits is observed on a screen^2,3^. The experiments demonstrate that the particle doesn’t form interference pattern when one detects which slit the particle passes through; the particle forms the interference pattern when one doesn’t detect which slit the particle passes through. These results show the principle of wave–particle duality^4,5^.

Feynman said that all of quantum mechanics can be gleaned from carefully thinking through the implications of this single experiment^6^, and he presented that if detectors were placed before each slit, the interference pattern would disappear^6^.

References^7,8^ used a single electron and biprism to show that each electron interferes with itself as predicted by quantum theory in 1974. Readers of Physics World voted the -electron version of the experiment as the most beautiful experiment^9^.

The double-slit experiment with electrons and real slits is eventually performed by the original scheme from Feynman, they emit electrons onto the double slits, and via collecting the transmitted electrons with a -electron detector, the double-slit interference pattern is shown^10^.

For antimatter, Ref.^11^ demonstrated a single particle interference. Photons, electrons, atoms and even some molecules are measured as a single pulse, the waves of these particles describe the probabilities of absorbing the particles at a specific place on the screen^12–14^.

The which-way experiment displays the complementarity principle that photons can show as either particles or waves, but cannot be observed as both at the same time^15^. In the history of quantum mechanics, despite the importance of the thought experiment, until the 1970s, technically feasible realizations of the thought experiment were presented^16^. In order to illustrate various aspects of complementarity, multiple experiments have been performed^17^.

In 1987, a performed experiment^18,19^ produced the results that the information could be gotten regarding a particle’s going which path.

Wave–particle duality of C60 molecules was investigated^20^, and Plasmon-Assisted Two-Slit Transmission was explored in Young's Experiment^21^.

Wheeler's delayed choice experiments showed that after a particle passes through the slits, extracting "which path" information at the slits can seem to retroactively alter its previous behavior^22^.

The new quantum universe is illustrated in Ref.^23^. The discovery that particles are discrete packets of energy with wave-like properties resulted in quantum mechanics dealing with atomic and subatomic systems. Furthermore, unification theory of classical statistical uncertainty relation and quantum uncertainty relation and its applications are shown^24^.

Although quantum theory has achieved the great successes in many aspects, there still is the hard puzzle of the single particle’s passing through Young's double slits simultaneously, the hard puzzle has been plaguing us up to now for decades, this paper wants to give the solution to the puzzle.

**Figure 1:**
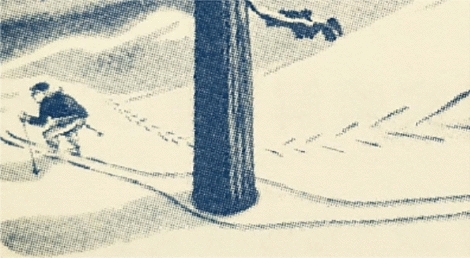
Coming from Charles Addams, The New Yorker, 1940.

For example: according to current quantum mechanics, any material can have wave-particle duality, therefore, the physical phenomenon in Fig. 1 could exist. But in real world, we don't see such a physical phenomenon, which is an interesting example of the fundamental puzzle in the theory of quantum mechanics. This paper wants to solve this kind of puzzle.

The arrangements of this paper are: Section “Studies on the puzzle of a particle’s passing through many slits simultaneously” gives studies on the puzzle of a particle’s passing through Young's Double Slits simultaneously; Section “Solutions to Wheeler’s delayed choice puzzle and puzzle of a particle’s passing double slits simultaneously by the physics processes of the exact quantum physics expressions” gives solutions to Wheeler’s delayed choice puzzle and puzzle of a particle’s passing double slits simultaneously by the physics processes of the exact quantum mathematical expressions; Section “New quantum control, quantum oscillation and their experiments” shows new quantum control, new quantum oscillation and their experiments; Section “Discussion, summary and conclusion” gives discussion, summary and conclusion.

## Studies on the puzzle of a particle’s passing through many slits simultaneously

In photoelectric effect, light waves cannot knock electrons out; and in a photon’s passing through many slit experiment, a photon cannot pass through many slits at the same time. Namely, the two physical processes, respectively, reflect one aspect of wave-particle duality of quantum particle. On the other hand, in photoelectric effect, photons can knock electrons out; in the many slit experiment, a photon light wave can pass through many slits at the same time. The two physical processes then are complementarily equivalent in wave-particle duality of quantum particle. That is, in wave-particle duality of quantum particle, the first and the second cases use the particle property and the wave property, respectively. Namely, a photon can show as either particle or wave, but cannot be observed as both at the same time for a physics process.

We now generally show them by exact deduction.

In 4-dimensional momentum representation of quantum theory, when considering wave function $\phi (\vec{p},E)$ of momentum representation, one has^25^

1 $$ \psi (\vec{r},t) = \frac{1}{{(2\pi \hbar )^{2} }}\int_{ - \infty }^{\infty } {} \phi (\vec{p},E)e^{{i(\vec{p} \cdot \vec{r} - tE)/\hbar }} d\vec{p}dE = \frac{1}{{(2\pi \hbar )^{3/2} }}\int_{ - \infty }^{\infty } {} \varphi (\vec{p},t)e^{{i\vec{p} \cdot \vec{r}/\hbar }} d\vec{p} $$

Equation (1) is a general Fourier transformation of $\, \phi (\vec{p},E)$ (about the plane wave energy E and momentum $\vec{p}$) from the four-dimensional momentum representation state vector $\, \phi (\vec{p},E)$ to the projection of the plane wave basic vector $e^{{i(\vec{p} \cdot \vec{r} - tE)/\hbar }}$ and making integration for getting $\, \psi (\vec{r},t)$, which make $\, \psi (\vec{r},t)$ have not only the characteristics of the probabilistic state vector of the particle but also the characteristics of the plane wave, i.e., make $\, \psi (\vec{r},t)$ have the state vector characteristics of wave-particle duality.

Because the momentum representation state vector $\, \phi (\vec{p},E)$ is nonlocal, it also reflects that the system has the global characteristics of momentum $\vec{p}$ and energy $E$, this global property can be the integrity of the particle, e.g., even including different physics qualities, e.g., spin, since the different qualities are not related to space coordinates.

Therefore, the expression (1) exactly shows wave-particle duality’s origin which displays that the wave property is originating from the plane wave part of the general Fourier expansion, and the particle property is originating from the general Fourier expansion coefficients with the particle’s global property even including different spins.

Therefore, we discover, for arbitrary particle, on an aspect, it propagates with the plane wave of the four-dimensional momentum $(\vec{p},E)$ as the propagation amplitude of the plane wave; on another aspect, it moves as a particle with the four-dimensional momentum $(\vec{p},E)$. Especially, when the expanding coefficients have different spins, it moves as a particle with both the four-dimensional momentum $(\vec{p},E)$ and the different spins, which are the new true physics and the new physical pictures, and uncover the corresponding expressions’ contributions of both wave part and particle part of wave-particle duality origin. Namely, Eq. (1) is the function of unified expression of wave-particle duality.

A little bit of philosophical insight on what this work means that the unified expression of wave-particle duality is just the superposition state of wave-particle duality, and the superposition state of wave-particle duality is physically real.

Furthermore, the infinite big momenta and energy show their corresponding to infinite big velocity in Eq. (1), and then the infinite big velocity is included, i.e., the wave function (1) of coordinate representation has the contribution of infinite big momentum or speed, namely, the wave function at any spatial and time points has the contributions from negative to positive infinite big momenta or speeds. Similarly, when we do the inverse Fourier transformation of Eq. (1) about whole spacetime coordinates, we find that the wave function of 4-dimentional momentum representation has the contributions of the whole 4-dimentional spacetime, i.e., the wave function at any 4-dimentialal momentum spatial point has the contributions from the whole spacetime. Thus, the above both cases just the reasons that Feynman path integral can be done in whole 4-dimentional spacetime or momentum space.

Using Eq. (1), we have wave function of momentum representation at time *t*

2 $$ \varphi (\vec{p},t) = \frac{1}{{(2\pi \hbar )^{1/2} }}\int_{ - \infty }^{\infty } {} \phi (\vec{p},E)e^{ - itE/\hbar } dE $$

On the other hand, using Huygens' Principle, one has the basic wave analysis:

Every point of a wave front may be considered the source of secondary wavelets that spread out in all directions with a speed equal to the speed of propagation of the waves. What this means is that when one has a wave, he can view the "edge" of the wave as actually creating a series of circular waves. These waves combine together in most cases to just continue the propagation, and in some cases there are significant observable effects. The wave front can be viewed as the line tangent to all of these circular waves^26^.

**Figure 2:**
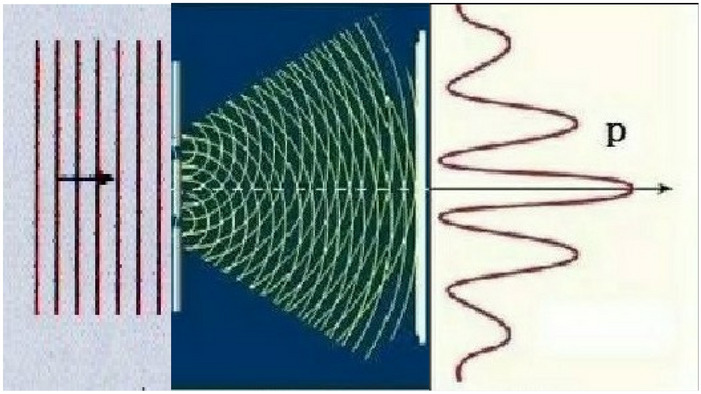
Interference of a particle plane wave in Young's double slit experiment.

Further using Eq. (1) and Huygens principle above, we have N subwave functions through N slits

3 $$ \psi (\vec{r}_{j} ,t) = \frac{1}{{(2\pi \hbar )^{2} }}\int_{ - \infty }^{\infty } {} \phi (\vec{p},E)e^{{i(\vec{p} \cdot \vec{r}_{j} - tE)/\hbar }} d\vec{p}dE = \frac{1}{{(2\pi \hbar )^{3/2} }}\int_{ - \infty }^{\infty } {} \varphi (\vec{p},t)e^{{i\vec{p} \cdot \vec{r}_{j} /\hbar }} d\vec{p} $$

where *j* = 1,2,……,*N*. No losing generality and for simplicity, taking *N* = 2 just shows the up slit and down slit, respectively, in Young's Double Slits in Fig. 2.

Therefore, Eqs. (1)–(3) can also be seen as a kind of expressions of Huygens principle. Consequently, these Fourier expansions physically imply new physics, and are not only just the mathematical tools.

The superposition density function of two subwaves is just Eq. (5) in Section “Solutions to Wheeler’s delayed choice puzzle and puzzle of a particle’s passing double slits simultaneously by the physics processes of the exact quantum physics expressions”, the interference terms of the two subwaves in Fig. 2 are just the third term and fourth term in Eq. (5).

These properties are exactly conforming to the plane wave properties of the single particle, thus a particle plane wave can simultaneously pass through N slits, for simplicity, Young's Double Slits in Fig. 2, Eq. (3) just generally give the both subwave functions that simultaneously pass through N slits, for simplicity, two slits s_1_ and s_2_ in Young's Double Slits, respectively.

The N subwave functions have the same amplitude $\phi (\vec{p},E)$ for some certain $\vec{p},E$, $e^{{i(\vec{p} \cdot \vec{r}_{j} - tE)/\hbar }}$ (*j* = 1, 2,……, N) are just N plane subwave functions in Eq. (3), and the N probabilistic wave functions in Eq. (3) integrate for ($\vec{p},E$) from negative infinite to positive infinite, i.e., having considered all possibility, which make the N expressions (3) exact.

The global property of a particle does not allow the single particle to simultaneously pass through N slits, for simplicity, Young's double slits, in reality, the interference of a particle wave is observed, which just show a particle wave simultaneously does pass the N slits, for simplicity, the double slits, but all theories up to now cannot solve the hard puzzle of a particle’s passing the N slits, e.g., Young’s double slits simultaneously.

## Solutions to Wheeler’s delayed choice puzzle and puzzle of a particle’s passing double slits simultaneously by the physics processes of the exact quantum physics expressions

The two subwaves (taking N = 2) from Huygens' Principle (see section “Studies on the puzzle of a particle’s passing through many slits simultaneously”) of a quantum Boson’s or Fermion’s Eq. (3) are entanglement each other, they may form interference of the subwaves, see Fig. 2, their entanglement wave function is

4 $$ \Psi (\vec{r}_{1} ,\vec{r}_{2} ,t) = c_{1} \psi (\vec{r}_{1} ,t) + c_{2} \psi (\vec{r}_{2} ,t),\;\;\;\;\left| {c_{1} } \right|^{2} + \left| {c_{2} } \right|^{2} = 1 $$

Using Eq. (4), we get the entanglement probabilistic density

5 $$ \rho (\vec{r}_{1} ,\vec{r}_{2} ,t) = \Psi^{\dag } (\vec{r}_{1} ,\vec{r}_{2} ,t)\Psi (\vec{r}_{1} ,\vec{r}_{2} ,t) = \left| {c_{1} \psi (\vec{r}_{1} ,t)} \right|^{2} + \left| {c_{2} \psi (\vec{r}_{2} ,t)} \right|^{2} + c_{1}^{\dag } \psi^{\dag } (\vec{r}_{1} ,t)c_{2} \psi (\vec{r}_{2} ,t) + c_{2}^{\dag } \psi^{\dag } (\vec{r}_{2} ,t)c_{1} \psi (\vec{r}_{1} ,t) $$

Equation (5) means that the single particle wave (1) passes through the double slits in Young's double slit experiment at time *t*, which are shown as the two subwaves that are entanglement subwaves (4).

When one measures that the single particle passes slit *s*_*1*_ and at time *t*_*1*_, then he must get $\psi (\vec{r}_{2} ,t_{1} ) = 0$ because of Eq. (4), consequently, using Eq. (5), we deduce

6 $$ \rho (\vec{r}_{1} ,\vec{r}_{2} ,t_{1} ) = \rho_{1} (\vec{r}_{1} ,t_{1} ) = \left| {c_{1} \psi (\vec{r}_{1} ,t_{1} )} \right|^{2} ,\int\limits_{{}}^{{}} {} \rho_{1} (\vec{r}_{1} ,t_{1} )d\vec{r}_{1} = 1 $$

Namely, there is no interference term in Eq. (6), which means that the interference disappears, i.e., the entanglement wave function density (5) is instantly collapsing into Eq. (6), which means that the single particle finally doesn’t simultaneously pass through Young's Double Slits by the disentanglement collapse in quantum mechanics.

When one measures that the single particle wave passes slit *s*_*2*_, the studies are similar.

When one doesn’t measure which way slit that the single particle wave passes, then Eq. (5) shows that there are interference terms, which means the single particle wave (1) simultaneously passes through Young's Double Slits by the probabilistic entanglement in quantum theory, which is shown as the two subwaves by Eqs. (4) and (5).

Wheeler's delayed choice puzzle: after a particle passes through the slits, extracting "which path" information at the slit in Young’s double slits can seem to retroactively alter its previous behavior, referencing to^22,27–29^ and its references.

Therefore, we find both entanglement and disentanglement for Wheeler's delayed choice experiment are quantum probabilistic entanglement and disentanglement, e.g., from Eqs. (4)–(6), further show that the quantum probabilistic disentanglement retroactively alters its previous passing through the slits’ behaviors from Eq. (5) with $\psi (\vec{r}_{2} ,t) \ne 0$ collapse to Eq. (6) with $\psi (\vec{r}_{2} ,t_{1} ) = 0$ in Wheeler’s delayed choice experiment with $t_{1} > t$. Therefore, this paper solves Wheeler’s delayed choice puzzle by the physics process of the exact quantum physics expressions (4)–(6).

We must point out that the distribution of the wave function $\psi (\vec{r}_{2} ,t) \ne 0$ is the distribution of the whole space, and this whole space automatically includes the space that the subwave returns from the original path *s*_*2*_ and enters through the other gap *s*_*1*_. Normally, the probability of taking this path is small, but since the particle state is measured to have passed through *s*_*1*_, which makes the particle state $\psi (\vec{r}_{{1}} ,t_{{1}} )$ equivalent to an attractor state, the probability of $\psi (\vec{r}_{2} ,t) \ne 0$ and all the other probabilities change to satisfy the physical reality of the particle having passed through *s*_*1*_'s gap at time *t*_1_.

This is the self-consistent requirement of probability conservation. Because the collapse of the wave function (4) is the collapse from all possible states to a measured state with density (6), and the measured state from the collapsed states is equivalent to the measured attractor state with density (6). So, we call the measured state through *s*_*1*_ as the measured attractor state with density (6). This collapse process is in fact the collapse process of all possible states towards the measured attractor state (or simply called as measured attractive state) with density (6).

These are the new physics of the collapse of all possible states (including, naturally, state $\psi (\vec{r}_{2} ,t) \ne 0$ that return backwards through the state of *s*_*2*_ and merge into the state of *s*_*1*_) to the measured attractor state $\psi (\vec{r}_{{1}} ,t_{{1}} )$. Consequently, we achieve the new key useful technology for changing the state evolution paths of quantum systems.

Because of using the usual entanglement theory, when the wave function of the system of the two subwaves $\psi_{1} (\vec{r}_{1} ,t)$ and $\psi_{2} (\vec{r}_{2} ,t)$ is expressed as

7 $$ \psi (\vec{r},t) = c_{12} \psi_{1} (\vec{r}_{1} ,t)\psi_{2} (\vec{r}_{2} ,t) $$

The system has no entanglement^27^; for the other cases, there are entanglements^27^. For example, Eq. (4) has entanglement from quantum probabilistic conservation.

Consequently, this paper finds the quantum probabilistic entanglement and disentanglement from Eqs. (4)–(6).

Therefore, the puzzle of Wheeler’s delayed choice is solved by the physics processes of the probabilistic entanglement and disentanglement expressions (4)–(6). Namely, after a particle wave passes through the slits, extracting "which path" information at one slit in Young’s double slits can seem to retroactively alter its previous behavior, which just originates from quantum disentanglement collapse that $\psi (\vec{r}_{2} ,t) \ne 0$ passed through the slit s_2_ instantly collapses to $\psi (\vec{r}_{2} ,t_{1} ) = 0$ and is incorporated into $\rho (\vec{r}_{1} ,\vec{r}_{2} ,t_{1} ) = \rho_{1} (\vec{r}_{1} ,t_{1} ) = \left| {c_{1} \psi (\vec{r}_{1} ,t_{1} )} \right|^{2}$, $\int\limits_{{}}^{{}} {} \rho_{1} (\vec{r}_{1} ,t_{1} )d\vec{r}_{1} = 1$. Because of having measured that the single particle has passed through the slit s_1_ at time *t*_1_ (> t, which is just the delayed choice’s measuring), that is, this quantum system collapses to the measured state when the system is measured at time *t*_1_.

In the same time, the puzzle of the single particle’s simultaneously passing through Young's Double Slits is also solved by the entanglement wave property (4) and entanglement wave function density (5) of wave-particle duality in quantum theory, namely, by the physics process of the exact quantum physics expressions (1), (4) and (5) in quantum probabilistic entanglement theory without the delayed choice measure.

## New quantum control, quantum oscillation and their experiments

**Figure 3:**
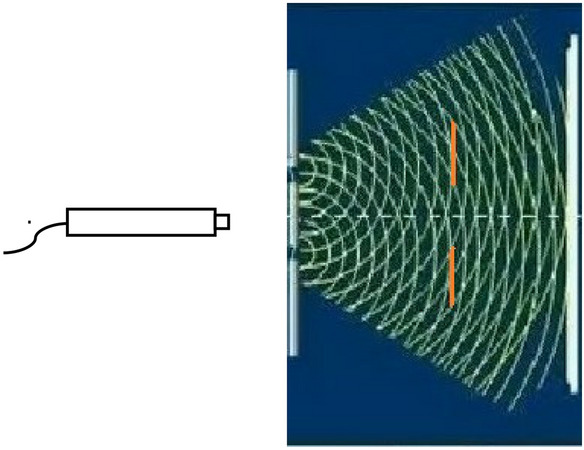
Interference of photon wave in the new quantum oscillation experiment.

For the quantum delayed choice experiment studied in the previous section, see Fig. 3, the left side of Fig. 3 is a single photon’s radiating gun that radiates a photon at every interval time T, two detectors further are mounted symmetrically on the up side and down side between Young's double slits and the screen, i.e., red line detector’s screen M1 is on the up side, red line detector’s screen M2 is on the down side. When the two detectors are closed, there are interference, see Fig. 3.

When M1 measures the particle passing through the up S1 slit, M1 is automatically closed (i.e., cancelling the measure, the system is restored to a new state), and M2 is automatically opened after time *t* (i.e., the system collapses again through measurement). When M2 detects a particle passing through the down slit S2, M2 is closed (to restore the system to a new state), and after time *t* opens M1 to measure (to cause the system to collapse again).

According to Wheeler’s delayed choice law and the collapse principle of measurement studied in the previous section, by adjusting the symmetric positions of M1 and M2 relative to the double slits, the interval time T and the time parameter *t*, the oscillation of the particle between paths M1 and M2 must appear, because the theory and the experiments are exactly based on the having been experimentally exactly tested bases of a measured state’s collapse and the returning to a new state after cancelling the measuring in quantum theory, i.e., the bases of the ingenious combinations of two Wheeler’s delayed choice experiments^1,2,6,23^. Through the new quantum control, it may modulate the new quantum oscillation of the particle by the generalized delayed choice experiment.

In particular, the interval time parameter *t* has very important physical implications. When the interval time *t* is equal to zero, i.e., one detector is turned off and the other detector is turned on at the same time. When the time *t* is not equal to zero, it reflects the degree of response of the quantum system, which is an important response parameter for quantum mechanics to describe the system. The size of the parameter *t* directly reflects the time effect of the physics reaction of the system. Based on the measured instantaneous collapse effect, this time response parameter should be very, very small, close to zero. We can automatically control the interval time T, the switch of the two detectors and the delayed detection time *t* of the two detectors by the program design of the computer connecting the two detectors and the single photon’s radiating gun.

For S2 measured by M1 and S1 measured by M2, the above method is equivalent.

For lots of the generalized delayed choice experiments about different bosons and fermions similar to Young's double slits above can be done. Especially, a similar delayed choice experiment for separating a beam light into two entangled beam lights through a biprism with properties of semi-permeable mirror and semi-reflex mirror can be done very similarly, which is written in the following paper because of the length constraint of this paper.

For a photon’s delayed choice experiment, when the new quantum control and the new quantum oscillation of above the research are realized very well, we are unable to see the photon signal on the screen because of the new quantum oscillation between the oscillation paths.

In this experiment, we first take the double slits away and close the two detectors, we can see the photon signal on the screen, which just show the photon’s particle property; and then we take the double slits backward the original position in Fig. 3 and close the two detectors M1 and M2, we can see the interference of photon wave on the screen, which just show the photon’s wave property; finally, all these investigations above mean that by adjusting the interval time T, the response time parameter *t* and the symmetrical positions of the two detectors M1 and M2 related to the double slits, so that we cannot see any photon signal on the screen in the new quantum oscillation experiment, then the new quantum control and the new quantum oscillation between the oscillation paths are realized very well.

Therefore, we get the objective criteria that the new quantum control and the new quantum oscillation between the oscillation paths are realized very well, and achieve the new controlling quantum particle’s oscillator being able to be installed in quantum communication network as the usual network and computer need the oscillator to work. All these are useful in quantum theory, quantum computer, quantum communications and so on. Especially, anyone working in this field can repeat the new quantum control and quantum oscillation experiments.

Utilizing the measured response parameter *t*, we are able to achieve the measured collapse velocity for the quantum system

8 $$ V_{collapse} = \frac{{L_{1} + \Delta + L_{2} }}{t}\mathop = \limits^{{L_{1} = L_{2} }} \frac{{2L_{1} + \Delta }}{t} $$

in which because detector M1 and detector M2 are symmetrically arranged about the double slits, e.g., see red lines in Fig. 3, then $L_{1} = L_{2}$ (are the distances between the two measure detectors and the double slits), $\Delta$ is the distance between the two slits. When time response parameter *t* $\to$ 0, $V_{collapse} \to \infty$. The collapse velocity, in the past, are unable to objectively be deduced.

Thus, this paper gives new quantum control, new quantum oscillation, their new realizable very important experiments and the new controlling quantum particle’s oscillator being able to be installed in quantum communication network and quantum computer from the new key useful technology for changing the state evolution paths of quantum systems in quantum theory, quantum computer and quantum communications.

## Discussion, summary and conclusion

Expression (1) shows wave-particle duality’s origin of displaying both the wave property originating from the plane wave part of the general Fourier expansion and the particle property originating from the general Fourier expansion coefficients with the particle’s global property and even including different spins etc., i.e., wave property and particle property both are expressed by general Fourier expansion in the same time, these are just the reasons that there exists wave-particle duality.

Thus, by using strict physical expressions, we give a physical representation of the wave- particle duality superposition state (1), and find that when measurements are made with devices that measure wave property or particle property, the wave-particle duality state collapses from the superposition state (1) of wave-particle duality to the manifestation state of wave property or particle property. So, these just explain why sometimes it's particle and sometimes it's wave.

The N subwaves of Eq. (3) are entanglement, they may form interference between subwaves, their entanglement wave function is Eq. (4) for N = 2.

When one measures that the single particle passes slit *s*_1_ at time $t_{{1}}$, thus he must get $\psi (\vec{r}_{2} ,t_{{1}} ) = 0$, because of quantum probabilistic entanglement Eq. (4). Consequently, using Eq. (5), we deduce that there is no interference term in Eq. (6), which means that the interference disappears.

When one doesn’t measure which slit that the single particle wave passes, then Eq. (5) shows that there are interference terms.

Therefore, we find that entanglement and disentanglement for Wheeler's delayed choice are, respectively, quantum probabilistic entanglement and quantum probabilistic disentanglement from Eqs. (4)–(6). Furthermore, this paper solves Wheeler’s delayed choice puzzle by the physics process of the exact quantum physics expressions (4)–(6).

In the photoelectric effect, one needs to think of light as particles, otherwise the electrons will be unable to be beaten out by light wave. That is, in the wave-particle duality, the particle property is only considered, wave property is not considered, and then the photoelectric effect is able to be explained. Similarly, in passing N slits, no losing generality for N = 2 Young's double slit experiment, the particle property is only considered, it is impossible for a photon to pass through two slits at the same time. But if we only think about wave property (because at this time, all particle properties are transformed into the particle’s wave properties with particle property as propagating coefficients that are just creation and annihilation operators of particles in quantum field theory), it is perfectly natural for the single photon wave to pass through the double slits at the same time.

In photoelectric effect and Young's double slit experiments, the single photon particle can hit electrons out, and the single photon wave can go through the N slits at the same time. Therefore, this paper discovers that the two cases are complementarily equivalent (i.e., the ability of the single photon to hit electron out is complementarily equivalent to the ability of wave of the single photon to pass through the N slits, e.g., Young's double slits at the same time in wave-particle duality, which is quantum physics of general bosonic systems).

Similarly, quantum particles such as electrons, neutrons, protons, photons and so on mean that wave of their single quantum particle can simultaneously pass through the N slits because at this time, all particle properties are transformed into the particle’s wave properties, rather than individual quantum particle may pass through the N slits at the same time, which is the new quantum physics of general Boson or Fermion particle systems.

This indicates that this effect is a quantum effect, because quantum effect has wave-particle duality. When considering wave property, we cannot consider particle property, because the whole quantum particle system is behaving like wave property (*Just as in the photoelectric effect, considering the particle nature of the system, people cannot consider wave property, otherwise the photoelectric effect cannot appear*). Namely, the single quantum particle can show as either particle or wave, but cannot be observed as the both at the same time at the same place for a physics process.

The single classical particle cannot simultaneously pass through the N slits, while wave of the single quantum particle can pass through the N slits at the same time, *these can be viewed as the objective criterion for distinguishing classical and quantum particles,* which is the new quantum physics of general particle systems.

Consequently, we discover the objective criterion for distinguishing classical and quantum particles. Thus, the objective criterion not only objectively gives the applicable realm of quantum mechanics that quantum particles satisfy, but also is key and very useful in quantum physics and science, which will greatly help us to distinguish, investigate and understand physics world and science. Therefore, for the single particle to simultaneously pass through the N slit experiment, needing to divide the studies into classical case and quantum case, the single classical particle cannot pass, while the single quantum particle wave may pass, which is the new quantum physics of general particle systems, then all the problems are solved.

For example, using the discovered objective criterion for classical and quantum particles, the puzzle of the example appearing in above Fig. 1 does not exist, which is the new quantum physics of general particle systems. Because as a person he is a macroscopic classical object, there is not such wave property to produce the physical phenomenon in Fig. 1.

The achieved theoretical results in this paper are also achieved experimentally by the experimental analysis and investigations^30^. By solving the delayed choice puzzle, we have obtained both the new measured attractor state concept and a new practical useful technique for controlling the changing evolutional paths of the wave function.

Therefore, this paper discovers new quantum physics, and gives solutions to Wheeler’s delayed choice puzzle and a particle’s simultaneously passing many slit puzzle by physics processes of exact quantum physics expressions, further shows new quantum control and new quantum oscillation.

This paper shows new very important feasible experiments of new quantum control and new quantum oscillation, and gets the objective criteria realizing the new quantum control and the new quantum oscillation, further achieves the new controlling quantum particle’s oscillator being able to be installed in quantum communication network and quantum computer as the usual network and computer need the oscillator to work. Furthermore, various quantum oscillators can be similarly constructed according to the similar principle^31^.

All these studies in this paper are very important and useful in quantum theory, quantum computer, quantum communications and so on, and are consistent with all the current physics theories and experiments. Consequently, all current articles, (text)books and concepts relevant to the results of this paper would be rewritten, updated and supplied. Following this paper, a lot of works can be done, which means that this paper would be widely propagated and largely cited.

## Data Availability

All data are included in this paper, if there are any other needs, we shall provide all the needs from our emails. Dr. C. Huang should be contacted if someone wants to request the data from this study.
